# Infiltrating ductal carcinoma breast with central necrosis closely mimicking ductal carcinoma in situ (comedo type): a case series

**DOI:** 10.1186/1752-1947-1-83

**Published:** 2007-09-08

**Authors:** Shahid Pervez, Hassan Khan

**Affiliations:** 1 Section of Histopathology, Department of Pathology and Microbiology, Aga Khan University Hospital, Karachi, Pakistan; 2 Medical College, Aga Khan University, Karachi, Pakistan

## Abstract

Here we present a series of infiltrative ductal carcinoma breast cases (infiltrative ductal carcinoma with central necrosis) so closely mimicking 'DCIS with central comedo necrosis' that on initial morphological analysis these foci of tumors were labeled as DCIS (high grade, comedo). However on further histological work up and by using immunohistochemistry (IHC) for myoepithelial markers it was later confirmed that these were foci of infiltrative ductal carcinoma breast with central necrosis. This case series gives the realization that a breast carcinoma may be partly or entirely DCIS like yet invasive. In such a dilemma IHC especially for assessment of myoepithelial lining is very useful to differentiate DCIS comedo from invasive carcinoma with central necrosis.

## Background

Proliferation of malignant epithelial cells within the ducts of the breast that show no light microscopic evidence of invasion through the basement membrane into the surrounding stroma is known as ductal carcinoma in situ (DCIS)[1]. Several morphologic patterns of DCIS are recognized, the most common of which are comedo, cribriform, solid, micro papillary and papillary. DCIS-comedo is diagnosed when at least one duct in the breast is filled and expanded by large, markedly atypical cells and has abundant central luminal necrosis [1]. It is well appreciated that infiltrating ductal carcinoma breast may mimic the diverse patterns of DCIS, the prototype of this being the infiltrating cribriform carcinoma [2].

Similarly here for the first time we present a series of infiltrative breast cancer cases (Infiltrative ductal carcinoma with central necrosis) so closely mimicking 'DCIS with central comedo necrosis' that on initial morphological analysis these tumors or foci were labeled as DCIS (high grade, comedo). However when axillary nodes were sampled, very similar morphologic pattern was seen in lymph node metastasis prompting immunohistochemical (IHC) studies on original biopsies with myoepithelial & basement membrane markers. This revealed a deficient/absent basement membrane & myoepithelial layer confirming the infiltrative nature of the initially diagnosed comedo type DCIS.

## Case Presentations

### Case 1

**Figure 1:**
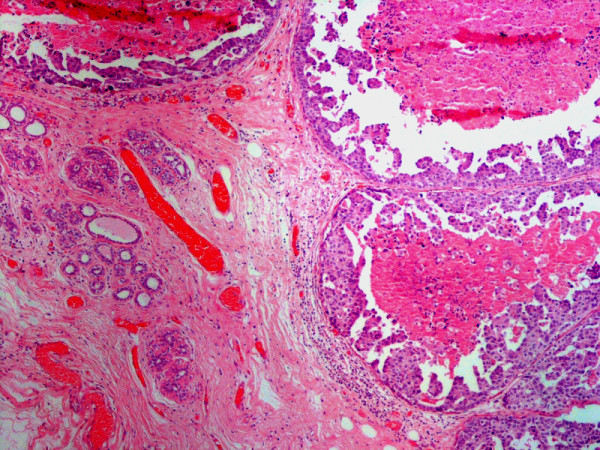
Breast lump showing infiltrating carcinoma breast with central necrosis initially interpreted as DCIS-comedo, H & E, Mag: 4×.

**Figure 2:**
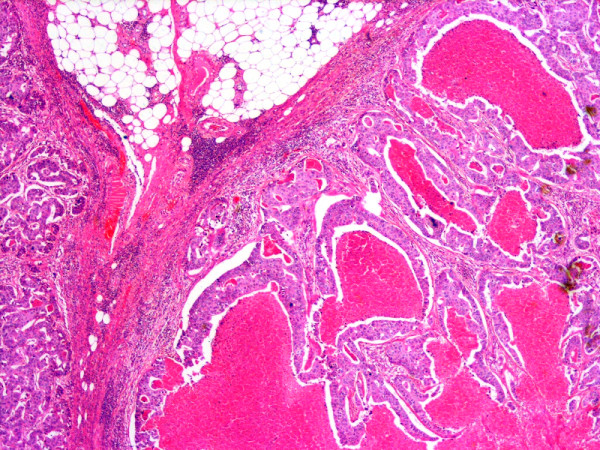
Axillary lymph node of the same patient showing metastatic ductal carcinoma breast with central necrosis closely mimicking DCIS with comedo necrosis, H & E, Mag: 2×.

**Figure 3:**
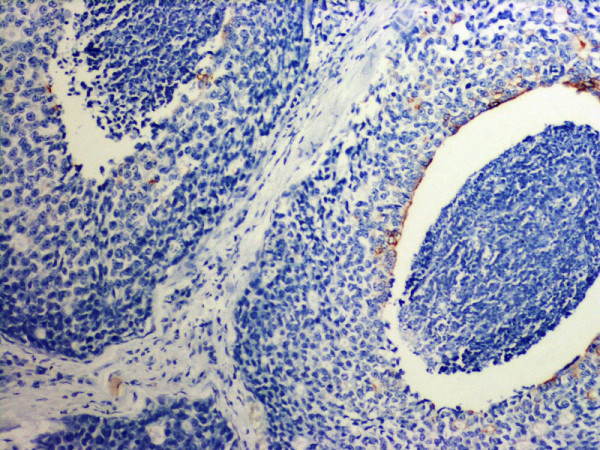
Same tumor as shown in Fig 1 stained with a cocktail of myoepithelial markers. Note absent myoepithelial layer consistent with the diagnosis of infiltrating ductal carcinoma with central necrosis. Mag: 10×.

A 56 year old woman presented with a breast lump of 3 × 2 × 1.5 cm in size submitted entirely. On histology it was reported as extensive DCIS comedo with no invasive component (Figure 1). On follow up examination axillary nodes became palpable and lymph node sampling was done. On histology one out of 14 lymph nodes showed 'metastatic breast carcinoma with central necrosis' closely mimicking DCIS comedo (Figure 2) in addition to some classic invasive foci. Breast lump slides were reviewed again with immunohistochemical staining for myoepithelium. All the foci which were interpreted as DCIS comedo lacked myoepithelial layer confirming the invasive nature of the tumor (Figure 3).

### Case 2

A 60 year old woman presented with a breast lump of 5.5 × 3 × 2.5 cm extensively sampled. On histology it was assessed as extensive comedo DCIS with occasional foci of invasion. However 35 out of 38 axillary lymph nodes showed extensive metastasis with pattern largely identical to what was reported high grade comedo DCIS. IHC for myoepithelial markers on original biopsy specimen again confirmed invasive nature of the DCIS comedo like foci with lack of myoepithelium.

### Case 3

A 45 year old woman's breast lump was reported as infiltrating ductal carcinoma (20%) with high grade DCIS Comedo (80%).Three out of twenty Lymph nodes showed extensive metastasis with similar DCIS Comedo like pattern. IHC again confirmed the invasive nature of the foci what was initially called as high grade DCIS. The size of the invasive component was recalculated for staging as IDC (70%); DCIS Comedo (30%).

### Case 4

A 42 year old woman presented with a breast lump of 2.5 × 2 × 2 cm reported as high grade comedo type DCIS. Lymph nodes were negative. Estrogen Receptor was positive, Progesterone Receptor was negative and HER2 by IHC was 3+ in what was called DCIS Oncologist denied the role of Herceptin as a part of therapy as HER2 expression was in DCIS only with no invasive component present. On review and IHC for myoepithelium, these foci lacked the myoepithelial layer and were relabeled as invasive carcinoma with central necrosis. Subsequently the patient was treated with Herceptin.

## Discussion

The risk factors for the development of invasive breast cancer and DCIS are similar. A further dilemma in the classification and histological analysis of DCIS is micro invasion. DCIS with micro invasion (DCISM) may also result in axillary lymph node metastases, whereas patients with DCIS should not, by definition, have axillary metastases. A higher suspicion for axillary metastases with DCISM can be obtained from the primary tumor characteristics. Statistically significant independent predictors of lymph node metastases in DCISM are comedo DCIS (*P *< 0.03) and the number of DCIS-involved ducts (*P *< 0.002) [1].

On pure morphological assessment a potential diagnostic trap is invasive ductal carcinoma with central necrosis. As the name indicates the tumor has a comedo DCIS like appearance and is likely to be diagnosed as DCIS comedo while in reality it is infiltrative breast carcinoma with central necrosis. This situation is identical to invasive cribiform carcinoma, a rare form of breast malignancy which very closely mimics cribriform DCIS [2]. The most important aspect of this concept is the realization that a breast carcinoma may be partly or entirely DCIS like, yet invasive. Recently a solid variant of invasive cribriform carcinoma is also described [3]. Similar morphologic patterns are also seen in salivary duct tumors, sweat gland carcinomas [4] and high grade prostatic adenocarcinomas. In case of the entire morphology having this feature, it is possible to report primary tumor as DCIS, following a conservative approach without further work up or axillary lymph node sampling. The other more common scenario is to incorrectly asses the size of the invasive component resulting in incorrect pTNM staging and management as pathological tumor size for classification (pT) is a measurement of only the invasive component [5].

**Figure 4:**
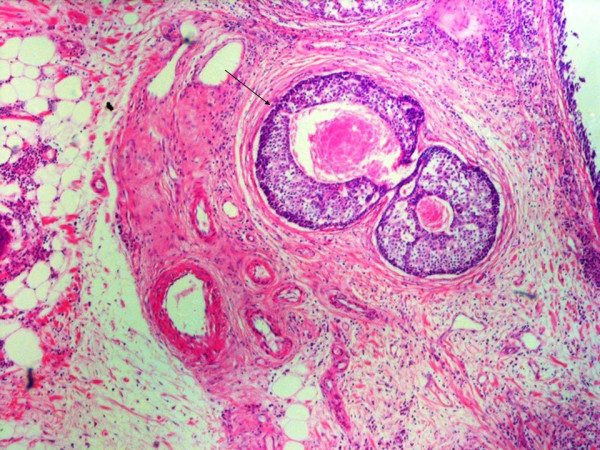
Foci of invasive ductal carcinoma breast with central necrosis. Note concentric stromal reaction around these foci (arrow), a helpful morphologic feature. H & E, Mag: 4×.

In such a dilemma IHC is very useful in assessment of invasion. In the ideal world invasive cancers are characterized by lack of both basement membrane and myoepithelial cells. However in the real world while invasive cancer lacks myoepithelial cells, some produce basement membrane components adding further to the confusion. Therefore for the assessment of DCIS and invasive comedo DCIS assessment of myoepithelial lining is most reliable. A number of myoepithelial markers including S-100, Alpha smooth muscle Actin, SMM – HC, Calponin and HMW-CK are available with different sensitivities and specificities. SMM-HC is thought to be the most specific while other though quite sensitive but are less specific. Some other myoepithelial markers include Maspin, CD10 and P63. Amongst these markers P63 is particularly useful as it stains the myoepithelial nuclei only with high sensitivity and specificity [6]. Myoepithelial antibody cocktail is another good choice [7]. With Actin one should be particularly careful not to confuse periductal myofibroblast staining as myoepithelial staining. In routine surgical pathology practice however it is not practical to do IHC for myoepithelium routinely on all such cases. One morphologic feature which we found useful on H & E was the concentric stromal reaction around these invasive foci (Fig 4). In addition irregular circumference of these invasive foci compared to true DCIS comedo was also helpful.

## Conclusion

The number of new breast cancer cases especially DCIS have increased multifold over the past decade owing to improved diagnostic testing especially mammography. This potential serious diagnostic error confusing high grade DCIS comedo with invasive carcinoma with central necrosis may be avoided by using IHC staining for myoepithelial markers and at times by subtle morphologic clues like stromal reaction.

## Competing interests

The author(s) declare that they have no competing interests.

## Authors' contributions

Shahid Pervez conceived the need to write up the cases, contributed to the work on the diagnostic dilemma related to these four cases, and reported histopathology of the cases including immunohistochemical work-up. Hassan Khan did the literature search and write up of the manuscript. Both authors reviewed the final manuscript.
